# Changes in six domains of cognitive function with reproductive and chronological ageing and sex hormones: a longitudinal study in 2411 UK mid-life women

**DOI:** 10.1186/s12905-020-01040-3

**Published:** 2020-08-14

**Authors:** Fanny Kilpi, Ana Luiza G. Soares, Abigail Fraser, Scott M. Nelson, Naveed Sattar, Sean James Fallon, Kate Tilling, Deborah A. Lawlor

**Affiliations:** 1 MRC Integrative Epidemiology Unit at the University of Bristol, Oakfield House, Oakfield Grove, Bristol, BS8 2BN UK; 2 Population Health Sciences, Bristol Medical School, Bristol, UK; 3 Bristol NIHR Biomedical Research Centre at University Hospitals Bristol NHS Foundation Trust and University of Bristol, Bristol, UK; 4 School of Medicine, Dentistry and Nursing, University of Glasgow, Glasgow, UK; 5 Institute of Cardiovascular and Medical Sciences, University of Glasgow, Glasgow, UK

**Keywords:** Menopause, Cognitive function, ALSPAC

## Abstract

**Background:**

There may be changes in cognitive function in women going through the menopause. The current evidence remains unclear, however, whether these changes occur over and above those of general ageing. We aimed to evaluate the potential impact of the menopause (assessed by reproductive age and hormone levels) on cognitive function in women in mid-life accounting for the underlying effects of ageing.

**Methods:**

The study was based on the follow up of women originally enrolled in pregnancy in a birth cohort when resident in the South West of England, UK between 1991 and 1992. Using up to three repeated measurements in 2411 women (mean age 51 at first assessment), we modelled changes in six cognitive function domains: immediate and delayed verbal episodic memory, working memory, processing speed, verbal intelligence and verbal fluency. The exposures of interest were reproductive age measured as years relative to the final menstrual period (FMP), chronological age and reproductive hormones (follicle-stimulating hormone (FSH), luteinizing hormone (LH) and anti-Müllerian hormone (AMH)).

**Results:**

Processing speed (− 0.21 (95% CI − 0.36 to − 0.06) standard deviation (SD) difference per 10 years since FMP), immediate verbal episodic memory (− 0.15 (95% CI − 0.35 to 0.06)) and delayed verbal episodic memory (− 0.17 (95% CI − 0.37 to 0.03)) declined with reproductive age. Reproductive hormones were not robustly associated with processing speed, but FSH and LH were both negatively associated with immediate (− 0.08 (95% CI − 0.13 to − 0.02) SD difference per SD difference in hormone level) and delayed verbal episodic memory (− 0.08 (95% CI − 0.13 to − 0.03)). There was little consistent evidence of cognitive function declining with menopause in other cognitive domains.

**Conclusions:**

Of the cognitive domains tested only verbal episodic memory declined both in relation to age since the menopause and in conjunction with the reproductive hormones that reflect the menopause. This decline was independent of normal ageing and suggests that the menopause is associated with a mild impact on this specific domain of cognitive function.

## Background

Understanding mechanisms responsible for cognitive changes during middle age may be key to developing treatments for the psychiatric and neurological conditions that are common in older adulthood. The menopause and its associated hormonal changes may be one such mechanism. Many women report experiencing changes in cognitive function, such as increased forgetfulness and poor concentration, during the menopausal transition [1]. Whether these alterations in cognitive function reflect the transition per se and its associated changes in reproductive hormones, or is related to symptoms of the menopause such as disruption of sleep by vasomotor symptoms, is unclear [2–4]. Furthermore, if there is any such impact of menopausal changes, whether specific aspects, i.e. ‘domains’, of cognitive function are adversely affected is uncertain (see Table 1). Other factors that occur in mid-life such as changing roles in family and work may also be involved. As evidence suggests age-related decline in cognitive function may start from early- to mid-40s in both women and men [9, 10], it is possible that menopause-attributed decline simply reflects chronological age-related change.

**Table 1:** Summary of the measures of cognitive function used in this study and the domains that they aim to assess

Domain	Test	Domain description	Brief description of what the test consisted of
Verbal episodic memory	Immediate logic memory test	A measure of ability to remember experienced events and other familiar, contextual information, immediately after events.	Told a ‘story’ in a standardised way (tape recording) then asked about key facts immediately after the ‘story’ was completed. Score reflects number of facts correctly remembered [5].
Verbal episodic memory	Delayed logic memory test	A measure of ability to remember experienced events and other familiar, contextual information, after a short delay.	As above but asked to recall key facts after undergoing other cognitive tests. Score reflects number of facts correctly remembered [5].
Working memory	Backward digit span test	A measure of ability of the ‘executive function’ to temporarily retain and manipulate information.	A test in which the tester says three digits (e.g. 9-1-7) and the participants were asked to repeat three digits backwards (i.e. 7-1-9). Score reflects number of trials passed [6, 7].
Processing speed	Digit symbol coding test	A measure of the speed at which the executive function can manipulate information.	Participants were asked to fill in symbols corresponding to certain numbers and given two minutes to complete as many entries as possible. Score reflects number of correct entries done in allocated time [7].
Verbal intelligence	Spot-the-word test	A measure of language-based general cognitive ability.	Participants were asked to identify the real word from a pair in which one is real and the other one is a made-up word. The score is the number of real words correctly identified [8].
Verbal fluency	Same letter word test	A measure which relates both to vocabulary as well as the speed at which information can be retrieved.	Participants were asked to list as many words as they could beginning with the letter ‘C’ in 1 min. This was repeated for the letters ‘F’ and ‘L’. The score reflects the number of correct words beginning with the letters that were freely recalled in the allocated time [6].

To understand whether cognitive function changes in relation to increasing reproductive age over and above any underlying change with chronological age requires repeated assessments of cognitive function in women across mid-life. Only one previous study has modelled reproductive age and chronological age simultaneously [11], and found evidence for a decline across years since final menstrual period (FMP), independently of chronological age, for verbal fluency, visuospatial abilities and episodic memory with up to three repeated measures in 193 women [11]. Other previous studies with repeated measurements of cognitive function studied across different menopausal stages suggest that verbal episodic memory, verbal fluency and processing speed may be impacted in peri- and post-menopause, but there is less compelling evidence of effects on working memory [2–4, 11–13]. The studies were generally small, but in the largest study to date, processing speed, verbal memory and working memory were tested up to four times between ages 45 and 63 (*N* = 1903) [2]. These domains of cognitive function were not found to differ by menopausal stage, but the authors suggested that transitioning through the menopause may be associated with a reduced ability to learn based on the lack of evidence of improvement with practice or ‘learning’ on repeats of the same cognitive tests.

In this study, we used rich longitudinal data to characterise how cognitive function is related to reproductive age (years since FMP) and reproductive hormones (follicle-stimulating hormone (FSH), luteinizing hormone (LH), and anti-Müllerian hormone (AMH)) as indicators of the menopausal transition. We use FSH, LH and AMH as biomarkers of the menopausal transition, because they show characteristic changes across the menopause [14–16]. Oestrogen was not measured because it is more variable in pre-menopause, and FSH and LH will reflect change in oestrogen over the menopausal transition. We explored whether any change by reproductive age was independent of changes related to chronological ageing, test practice (‘learning’) effects and potential confounders. A priori we considered that showing associations of both reproductive age and reproductive hormones with any domain of cognitive function would provide more robust evidence of a likely impact than associations with just one of these. In secondary analyses, we sought to replicate the analysis that menopause may be associated with a reduced ability to learn [2].

## Methods

### Study participants

We used data from the mothers of the Avon Longitudinal Study of Parents and Children (ALSPAC) birth cohort. Full details of the study have been previously reported [17, 18]. ALSPAC enrolled 14,541 pregnancies in the South West of England (around the city of Bristol) with an expected delivery date between 1st April 1991 and 31st December 1992. The participating families have been followed up through to the current day [17]. Please note that the study website (http://www.bristol.ac.uk/alspac/researchers/our-data/) contains details of all the data and interview guides that are available through a fully searchable data dictionary and variable search tool.

**Fig. 1:**
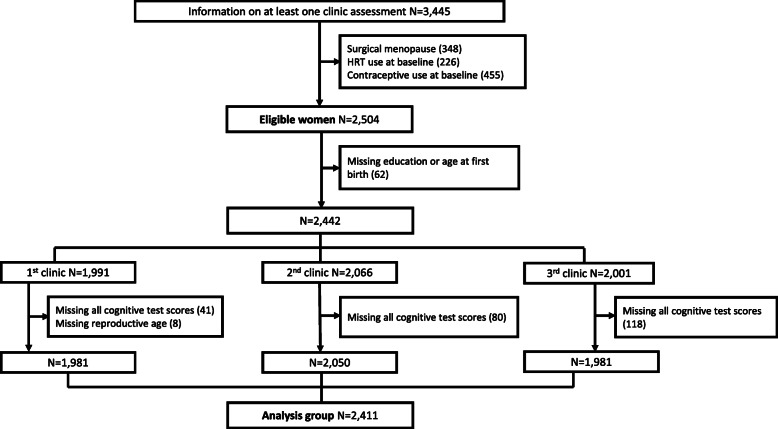
Participant flow into eligible and analysis groups, 2011–2015

In 2009–2011, all mothers still engaged with the study (*N* = 11,264) were invited to a follow up assessment clinic, with 4834 (43%) of invited women attending. The participating women were older and more educated than the original sample recruited in pregnancy [18]. A further three follow-up assessment clinics, each successively 1 to 2 years apart, were undertaken focusing on women who were pre-menopausal in the initial clinic and therefore likely to go through the menopausal transition during the subsequent three assessments, reflecting the aim to explore social, lifestyle, health and biological changes as women go through the menopausal transition [19]. This study is restricted to these three later clinics in which cognitive function tests were administered. Figure 1 describes the participant flow into the analyses. Women were included irrespective of whether they changed through one or all three of the menopausal stages of pre-, peri- and post-menopause as our primary exposures were not these categories but reproductive age and hormones. Women who had undergone surgical menopause at baseline or follow up were excluded, as were women reporting using hormone replacement therapy (HRT) or hormonal contraception at baseline, so that the focus was on changes occurring across a natural menopause. Observations for women who reported using HRT or hormonal contraception in follow up were also censored at the last point before reported use. The analysis sample consisted of 2411 women with 1386 women participating in all three assessment clinics. A majority of the participants (97%) were White British.

### Reproductive age

Women were asked a detailed set of questions about the date of their last menstrual period and the regularity of their menses by interview at each assessment clinic. These questions were designed to be able to categorise participants into Stages of Reproductive Aging Workshop (STRAW) categories [15]. FMP could be identified when at least 1 year of amenorrhea had occurred since the date of the last menstrual period. Using this information, reproductive age was calculated retrospectively using years since FMP and coded as zero when women were pre-FMP. Reproductive age could not be measured before FMP due to the relatively small number of women having their FMP during the study follow up. A binary variable on whether the woman had reached their FMP was also determined for each assessment clinic.

### Hormones

Levels of FSH, LH, and AMH were assessed from fasting samples in women at the three assessment clinics without restrictions on which day in the menstrual cycle the participants were at the time of blood sampling. Women were instructed to fast overnight or for at least 8 h before the clinic visit, and the blood samples were processed within 4 h and stored at − 80 °C until thawed for hormonal analyses (with no previous thaw-freeze cycles). Serum FSH, LH and AMH were measured with a Roche Elecsys modular analytics Cobas e411 using an electrochemiluminescence immunoassay. The AMH assay used was the fully automated Elecsys AMH Plus immunoassay from Roche Diagnostics [20].

### Menopausal stage

STRAW criteria [15], using the date of the last menstrual period and the regularity of menses, were used to categorise women into menopausal stages [19]. In this study, we condensed the more detailed categories into (i) pre-menopausal (reproductive, STRAW categories − 5 to -3a), (ii) peri-menopausal (menopause transition and first year post-menopause, STRAW − 2, − 1 and + 1a) and (iii) post-menopausal (from second year post-menopause, STRAW +1b to + 2).

### Cognitive tests

Six different cognitive tests were administered at each of the three assessment clinics according to a standardised protocol to assess specific domains of cognitive function (see Table 1). Higher scores on each test reflect better cognitive function.

### Confounders

We adjusted for (1) educational attainment, as defined by the highest attained qualification (i) Certificate of Secondary Education (CSE), ordinary- (O-) level or vocational certificate (qualifications usually obtained at age 16, the UK minimum school leaving age when these women were at school), (ii) Advanced A-level (usually taken at 18 years) or (iii) university degree, and (2) age at first pregnancy. Information on both were obtained by questionnaire when the women were first recruited.

As the period between each of the assessments was 1 to 2 years, practice effects may have occurred in cognitive test performance. That is, performance may have improved, or an age-related decline be somewhat masked, as a result of familiarity with the test. We accounted for this in our analyses with a (3) time-varying continuous variable detailing the number of previous testing occasions. In addition, we adjusted for (4) the fieldworker who had administered the test to reduce any potential variation in performance related to how the tests were administered.

### Statistical methods

Descriptive statistics were calculated and cognitive test scores at the first assessment clinic were examined by menopausal stage using analysis of variance.

#### Main analyses

Full details of the strategy for the main analyses, including details of all multilevel models, are provided in Supplementary Text (Additional file 1). Briefly, we used multilevel linear regression models to examine: (i) change in cognitive function domains by reproductive age (years since FMP) and chronological age and compare the contributions of each of these and (ii) the association of standardised LH, FSH and AMH levels (using mean and standard deviation (SD) from first assessment clinic, having replaced undetectable LH and AMH levels with 0.1 mIU/ml and 0.01 ng/ml respectively) with cognitive function. Multilevel models allow all women with at least one cognitive function assessment to be included in analyses under a missing-at-random (MAR) assumption and take account of the correlation between repeated measurements. As we only had up to three measurements in each woman, we had to assume any change with reproductive or chronological age or association with hormones were linear. We modelled each cognitive function domain in SD units, using the mean from the first assessment clinic and the estimated between-individual SD derived from the fully adjusted model.

The Bayesian Information Criteria (BIC) was used to assess and compare how reproductive and chronological age explained variation in cognitive function. The main models were adjusted for fieldworker effects, practice effects, chronological age, education and age at first pregnancy. To assess associations of reproductive hormones (FSH, LH and AMH) with cognitive function, each was included as a time-varying exposure in separate models, with the results reflecting the difference in cognitive function between women with one SD difference in hormone level at any given age.

Lastly, we studied differences in the extent of improvement in cognitive function by practice at pre-, peri- and post-menopause. We tested whether the interaction between practice effects (with a random slope) and menopausal stage improved model fit in a model including chronological age, education and age at first pregnancy using log likelihood tests.

#### Sensitivity analyses

We compared baseline cognitive function scores by the duration of follow-up time available to examine whether results may have been biased by loss to follow-up. We also repeated the main analyses in a sample restricted to women who participated in all three clinics. All analyses were conducted in Stata 15.1 (StataCorp, Texas, US) and MLwIN version 3.01 using command ‘runmlwin’ [21].

### Ethical approval

Ethical approval for the data collection was obtained from the ALSPAC Ethics and Law Committee and the Local National Health Service Research Ethics Committees. Informed written consent for the use of data collected via questionnaires and clinics was obtained from participants. Consent for biological samples has been collected in accordance with the Human Tissue Act (2004).

## Results

### Participant characteristics

Table 2 details the characteristics of the participants by assessment clinic. The mean age of the women at the first assessment was 51 years (SD 4.4, Table 2), and the average age at menopause of the women was 50 (SD 3.8). The age range of women during the study was between 36 and 66 years. 38% of women were identified as pre-menopausal at the first assessment, decreasing to 20% by the last. With the exception of verbal intelligence, mean performance on all cognitive tests increased modestly across the three assessments.

**Table 2:** Characteristics of participants at the three assessment clinics (*N* = 2411)

	Clinic 1	Clinic 2	Clinic 3
	*N* = 1981	*N* = 2050	N = 1981
**Age, mean (SD)**	50.9 (4.4)	52.2 (4.4)	53.3 (4.4)
**Years since final menstrual period, mean (SD)**	1.90 (3.4)	2.54 (3.8)	3.19 (4.3)
**Menopausal stage**			
Pre-menopause, N (%)	749 (38%)	544 (27%)	394 (20%)
Peri-menopause, N (%)	532 (27%)	532 (26%)	479 (24%)
Post-menopause, N (%)	659 (33%)	807 (39%)	925 (47%)
Unable to determine, N (%)	41 (2%)	167 (8%)	183 (9%)
**Cognitive function score**			
Immediate verbal episodic memory, mean (SD), sample range 3–25	15.6 (3.6)	16.0 (3.3)	16.6 (3.3)
Delayed verbal episodic memory, mean (SD), sample range 0–25	14.4 (3.8)	15.0 (3.6)	15.9 (3.5)
Working memory, mean (SD), sample range 2–14	7.1 (2.4)	7.2 (2.3)	7.4 (2.3)
Processing speed, mean (SD), sample range 10–133	80.7 (13.8)	82.8 (13.6)	83.7 (14.0)
Verbal intelligence, mean (SD), sample range 0–60	44.1 (7.7)	44.2 (7.3)	44.2 (7.4)
Verbal fluency, mean (SD), sample range 0–98	43.2 (12.0)	45.1 (12.5)	45.8 (12.6)
**Education**			
CSE / Vocational / O-level, N (%)	925 (47%)	977 (48%)	930 (47%)
A-level, N (%)	597 (30%)	618 (30%)	599 (30%)
Degree, N (%)	459 (23%)	455 (22%)	452 (23%)
**Age at first pregnancy, mean (SD)**	26.7 (4.7)	26.6 (4.8)	26.6 (4.7)
**Reproductive hormones**			
AMH (ng/ml), median (IQR)	0.01 (0.01–0.17)	0.01 (0.01–0.08)	0.01 (0.01–0.03)
FSH (mIU/ml), median (IQR)	35.9 (7.4–74.2)	58.0 (11.7–87.9)	63.8 (18.7–88.9)
LH (mIU/ml), median (IQR)	24.4 (7.3–40.0)	31.5 (10.6–44.0)	31.8 (15.4–43.2)

*AMH* anti-Müllerian hormone, *FSH* follicle-stimulating hormone, *IQR* interquartile range, *LH* luteinizing hormone, *SD* standard deviation

Table 3 shows cognitive test scores by menopausal stage in women participating in the first assessment clinic. There were small differences in mean levels of processing, verbal intelligence and verbal fluency across categories of menopausal stage. For processing speed levels were highest in pre-menopausal women (mean 82.5, SD 13.8), intermediate in those who were peri-menopausal (80.6, SD 13.8) and lowest in those who were post-menopausal (78.7, SD 13.5). For verbal intelligence and verbal fluency, the direction of association was the opposite, with levels being lowest in pre-menopause and highest in post-menopausal women. Mean levels of immediate and delayed verbal episodic memory and working memory were similar across the menopausal stages.

**Table 3:** Cognitive function at the first assessment clinic by menopausal stage (*N* = 1894–1909)

	Mean (SD) score by menopausal stage			***P***-value*
	Pre-menopause	Peri-menopause	Post-menopause	
**Immediate verbal episodic memory**	15.6 (3.4)	15.7 (3.6)	15.5 (3.7)	0.41
**Delayed verbal episodic memory**	14.4 (3.6)	14.5 (3.9)	14.3 (3.9)	0.55
**Working memory**	7.0 (2.4)	7.2 (2.3)	7.2 (2.4)	0.11
**Processing speed**	82.5 (13.8)	80.6 (13.8)	78.7 (13.5)	< 0.001
**Verbal intelligence**	42.3 (7.7)	44.6 (7.4)	45.7 (7.4)	< 0.001
**Verbal fluency**	42.1 (11.9)	43.8 (11.8)	43.8 (12.3)	0.01

*P-value from an ANOVA test for trend. Cognitive test scores by menopausal stage in women participating in the first assessment clinic

### Main results

#### Reproductive age and cognitive function

Supplementary Table 1 (Additional file 1) details the results of the different models for cognitive function by reproductive and chronological age. These suggest that both reproductive age and chronological age contribute to change in cognitive function. In simpler models, chronological age tended to have a stronger relationship with cognitive function compared with reproductive age, except for processing speed.

**Fig. 2:**
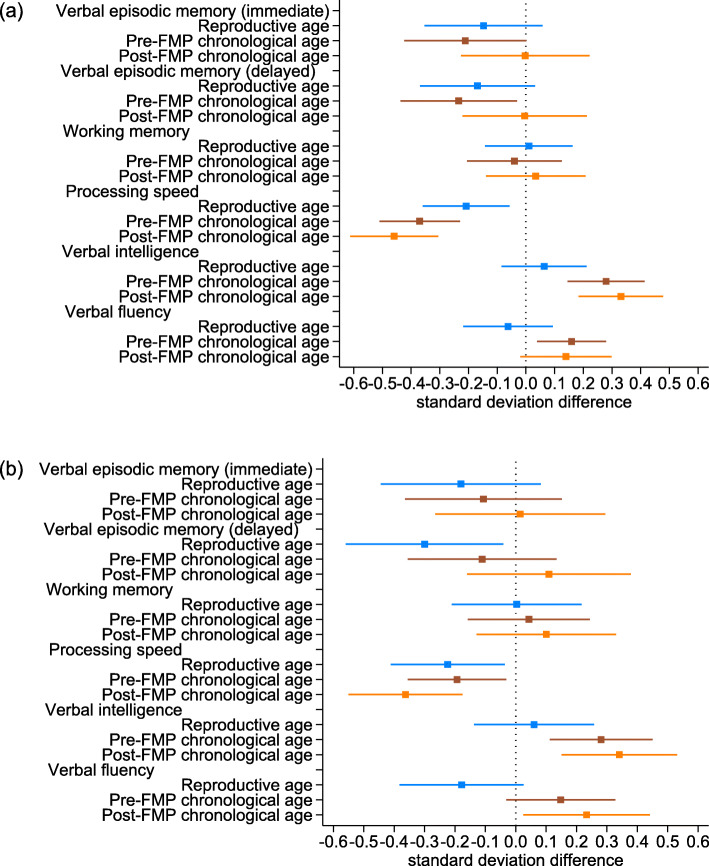
Difference in standardised cognitive function by 10 years greater reproductive and chronological age. **a** Main analyses including all women with at least one measure of cognitive function (*N* = 2402–2408). **b** Sensitivity analyses restricted to women with measures of cognitive function at all three time points (N = 1385–1386). Note: Reproductive age is measured as years since final menstrual period (FMP) and chronological age is centred at age 50 and in interaction with whether the observation is before and after FMP. Analyses adjusted for practice effects (baseline assuming no practice effects), fieldworker effects, education (baseline CSE/vocational/O-level) and age at first pregnancy (centred at 26)

The fully adjusted associations of reproductive and chronological age with cognitive function are presented in Fig. 2a (detailed results of different models are shown in Supplementary Table 1, Additional file 1). Reproductive age was negatively associated with immediate (SD difference − 0.15, 95% CI − 0.35 to 0.06) and delayed (− 0.17, 95% CI − 0.37 to 0.03) verbal episodic memory and processing speed (− 0.21, 95% CI − 0.36 to − 0.06), though for the former confidence intervals were wide and consistent with no difference. Working memory (0.01, 95% CI − 0.14 to 0.16), verbal intelligence (0.06, 95% CI − 0.08 to 0.21) and verbal fluency (− 0.06, 95% CI − 0.22 to 0.09) changed little with reproductive age. Associations of chronological age with cognitive function were more complex, with positive associations with verbal intelligence (0.28, 95% CI 0.15 to 0.41) and verbal fluency (0.16, 95% CI 0.04 to 0.28), negative associations with both immediate (− 0.21, 95% CI − 0.42 to 0.00) and delayed (− 0.23, 95% CI − 0.44 to − 0.03) verbal episodic memory and processing speed (− 0.37, 95% CI − 0.51 to − 0.23), but for former only before FMP, and a largely null association with working memory (− 0.04, 95% CI − 0.21 to 0.12).

#### Reproductive hormones and cognitive outcomes

**Fig. 3:**
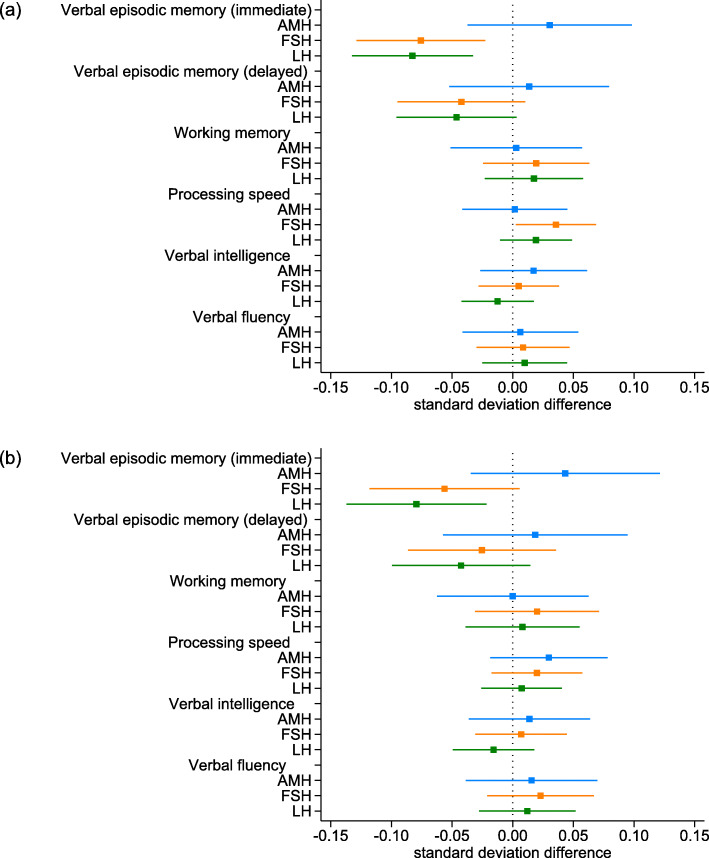
Difference in standardised cognitive function by standardised levels of anti-Müllerian, follicle-stimulating and luteinizing hormone. **a** Main analyses including all women with at least one measure of cognitive function (*N* = 2209–2213). **b** Sensitivity analyses restricted to women with measures of cognitive function at all three time points (*N* = 1348). Note: AMH: anti-Müllerian hormone, FSH: follicle-stimulating hormone, LH: luteinizing hormone. Analyses are adjusted for age (centred at 50), practice effects (baseline assuming no practice effects), fieldworker effects, education (baseline CSE/vocational/O-level) and age at first pregnancy (centred at 26)

The results for the fully adjusted associations of FSH, LH and AMH with cognitive function are shown in Fig. 3a (detailed results of different models are shown in Supplementary Table 2, Additional file 1). FSH and LH, which increase around menopause, were negatively associated with immediate (− 0.08, 95% CI − 0.13 to − 0.02, and − 0.08, 95% CI − 0.13 to − 0.03, respectively) and delayed (− 0.04, 95% CI − 0.09 to 0.01, and − 0.05, 95% CI − 0.10 to 0.00, respectively) verbal episodic memory, and FSH was positively associated with processing speed (0.04, 95% CI 0.00 to 0.07).

### Sensitivity analysis

Women who were lost to follow up had lower baseline scores on most of the tests, but notably baseline immediate and delayed verbal episodic memory scores did not differ by loss to follow up (Supplementary Tables 3 and 4, Additional file 1). When we restricted analyses to women who participated in all three assessment clinics, the associations of reproductive age with cognitive function were similar to those of the main analyses (Fig. 2b and Supplementary Table 5, Additional file 1). However, in these analyses there was a stronger decrease in delayed verbal episodic memory (− 0.30, 95% CI − 0.56 to − 0.04) and verbal fluency (− 0.17, 95% CI − 0.37 to 0.02) with increasing reproductive age. Similarly, associations of reproductive hormones with cognitive function in the restricted sample of women with data from all three assessment clinics were broadly similar to the main analyses (Fig. 3b and Supplementary Table 6, Additional file 1).

### Secondary analyses: differential practice effects

Table 4 presents results for differential practice effects in the main analysis participants. There was statistical support for a practice effect on processing speed, such that peri-menopausal women had lower mean scores at their first assessment (SD difference − 0.02, 95% CI − 0.13 to 0.08), but a slightly greater improvement by practice than pre-menopausal women (interaction term 0.07, 95% CI − 0.01 to 0.15) (P_interaction_ = 0.0044). For immediate (− 0.09, 95% CI − 0.24 to 0.06) and delayed (− 0.14, 95% CI − 0.29 to 0.01) verbal episodic memory, post-menopausal women had lower baseline scores than pre-menopausal women but a greater improvement in scores with repeated testing (interaction terms 0.09, 95% CI 0.00 to 0.18, and 0.09, 95% CI 0.01 to 0.18, respectively), though there was no strong statistical support for an interaction (P_interaction_ = 0.0581 and 0.1119, respectively). For other outcomes, change with practice was similar across the menopausal stages.

**Table 4:** Cognitive function around menopause with menopausal stage and number of previous testing occasions (N = 2402–2408)

	Verbal episodic memory		Working memory	Processing speed	Verbal intelligence	Verbal fluency
	Immediate logical memory	Delayed logical memory	Backward digit span	Digit symbol coding	Spot-the-word	Same letter word
	SD difference (95% CI)	SD difference (95% CI)	SD difference (95% CI)	SD difference (95% CI)	SD difference (95% CI)	SD difference (95% CI)
**Previous testing occasions**	0.32 (0.25, 0.39)	0.39 (0.32, 0.46)	0.12 (0.06, 0.17)	0.24 (0.20, 0.28)	−0.02 (−0.06, 0.02)	0.14 (0.09, 0.18)
**Menopausal stage**						
Pre-menopause	(ref)	(ref)	(ref)	(ref)	(ref)	(ref)
Peri-menopause	0.05 (−0.08, 0.19)	− 0.00 (− 0.13, 0.13)	−0.02 (− 0.13, 0.08)	−0.10 (− 0.18, − 0.03)	0.03 (− 0.05, 0.10)	0.08 (− 0.01, 0.16)
Post-menopause	−0.09 (− 0.24, 0.06)	−0.14 (− 0.29, 0.01)	−0.03 (− 0.15, 0.09)	−0.05 (− 0.14, 0.04)	0.02 (− 0.07, 0.11)	−0.02 (− 0.12, 0.08)
**Interaction**						
Previous testing occasions X peri-menopause	−0.01 (− 0.12, 0.09)	0.05 (− 0.05, 0.15)	0.07 (− 0.01, 0.15)	0.06 (− 0.00, 0.12)	0.00 (− 0.05, 0.06)	−0.01 (− 0.08, 0.05)
Previous testing occasions X post-menopause	0.09 (− 0.00, 0.18)	0.09 (0.01, 0.18)	0.05 (− 0.02, 0.12)	−0.03 (− 0.08, 0.02)	0.02 (− 0.02, 0.07)	0.04 (− 0.02, 0.09)

Analyses are conducted in the main analysis participants, with adjusted for number of previous testing occasions (practice effects), menopausal stage, fieldworker effects, age (centred at 56), education (baseline CSE/vocational/O-level) and age at first pregnancy (centred at 26)

## Discussion

### Main findings

After taking account of chronological age, learning effects and socioeconomic confounding, we found that the menopause, assessed by reproductive ageing and hormones, had little impact on most cognitive domains, with the exception of immediate and delayed verbal episodic memory. If the menopause has an adverse effect on cognition above and beyond chronological age, we might have expected consistent results for all outcomes and consistency between reproductive age and hormones. However, we found consistency between reproductive age and reproductive hormones only for the two verbal episodic memory tests, providing some evidence that that this cognitive domain may be influenced modestly by the menopause, whilst highlighting that most aspects of cognitive function were not.

In order to give a sense of the size of the estimated associations of reproductive age with cognitive function, we compared our results with those of an independent UK study (Whitehall II) that examined ageing and cognition [10]. The decrease in verbal episodic memory by reproductive age in our study (− 1.28% for immediate and − 1.58% for delayed verbal episodic memory per 10 years since FMP) were approximately half the size of equivalent results by chronological age in the Whitehall II study, in which women aged between 45 and 49 were estimated to have a decline of 2.5% in verbal episodic memory (measured with a 20 word recall test) over the next 10 years (full details of how we harmonised our results to those from Whitehall II are provided in Supplementary Text and full results in Supplementary Table 7, Additional file 1).

### Study strengths and limitations

ALSPAC is one of a limited number of longitudinal studies with in-depth assessments of cognitive function, hormones and menstrual history in women around the time of the menopause [2–4, 11–13], and to our knowledge the largest study to date with such data. It is also perhaps the most comprehensive in accounting for potential confounders. The longitudinal study design enabled us to observe any potential change within women as they initially entered the menopausal transition and in postmenopausal years, though the closely spaced testing occasions also created practice effects. The effects of age may also have been partly confounded by socioeconomic resources, because older women in this study were more likely to be mothers who had had children later, which is generally associated with higher socioeconomic resources [22]. We adjusted for education and age at first pregnancy to control for this confounding.

Multilevel models allow all women to be included if they have at least one measure under the assumption that data are missing at random (i.e. that associations do not differ in those who have fewer repeated measurements). Whilst there was some evidence that cognitive function at baseline was lower amongst those lost to follow up, analyses restricted to participants with all three repeated measurements did not differ substantively from the main analyses, suggesting that selection bias is not a major concern. We fit linear spline multilevel models because cognitive function was only measured on three occasions and fitting non-linear change with reproductive or chronological age, for example, using fractional polynomial or other ‘smoothing’ methods, was not possible [23, 24]. Based on these results we cannot therefore determine whether the decline in cognitive function by reproductive age is permanent or transient. We also could not model reproductive age before the FMP because relatively few women experienced their FMP during the follow up (*N* = 164). Despite ours being one of the largest studies to explore these associations, further larger studies would be valuable.

We were able to look for consistency between change in cognitive function in relation to years since the FMP and the associations with reproductive hormones, which has not been done previously. FSH, LH, AMH show characteristic changes across stages of reproductive ageing and may be used as more precise biomarkers of reproductive age than self-reported FMP. They also capture changes that occur earlier in the menopausal transition than after the FMP. Although we did not have information on change in oestrogen in these women, evidence from randomised control trials of HRT suggests there is no major influence of HRT on cognitive function in young post-menopausal women [25–29]. Previous studies show a mirror image of increasing FSH and LH and decreasing oestrogen for about 2 years before and after the FMP and thereafter plateauing, and FSH has been described as the ‘best classifier’ for diagnosing the natural menopause [14, 30, 31]. AMH, in turn, is a biomarker of ovarian function and may be the most stable and accurate predictor of time to menopause [16].

### Comparisons with previous studies

Our finding of a decrease in immediate and delayed verbal episodic memory with menopause is consistent with evidence by menopausal stage from previous longitudinal studies such as analyses of the Study of Women’s Health Across the Nation (SWAN) [2, 4, 13]. Few studies have modelled change in cognitive function by years since FMP, but one study suggested small declines in episodic memory, as well as verbal fluency and visuospatial abilities [11]. An analysis of SWAN data demonstrated declines in processing speed and a small decline in delayed verbal episodic memory by years relative to FMP, when adjusted for age at FMP and covariates (education, financial hardship, race, language, hormone therapy, oophorectomy, diabetes, menopause symptoms, number of assessments) [32]. The decline in processing speed that we observed by reproductive age was also consistent with its decline across menopausal stages in previous longitudinal studies [3, 4], although we did not find decrements in practice effects or learning in the menopausal transition as suggested by an another analysis of the SWAN data [2].

The one previous observational longitudinal study of hormonal changes and cognitive function that we are aware of showed associations between FSH and LH and the four administered cognitive tests (immediate and delayed verbal recall, processing speed, and sensorimotor processing speed) in crude models, but not when adjusted for covariates (age, race, education, and BMI) in a sample of 403 women [4]. FSH, LH and AMH show characteristic changes during the menopause, in addition to the variability of FSH and LH levels in the menstrual cycle [14, 15]. In our study, overall, there was a lack of evidence for associations of reproductive hormones with most cognitive domains, but FSH and LH, which increase during the menopause transition from around 2 years before FMP to 2 years after [14, 15], were associated with a lower score in verbal episodic memory in adjusted models, which is consistent with the inverse association of reproductive age with these measurements. It is possible that the associations with FSH and LH reflect the effects of declines in oestrogen at menopause, but we could not test this with the data.

## Conclusions

Many women report experiencing cognitive symptoms during menopause. In this large cohort of women, we show that there may be a modest decline in immediate and delayed verbal episodic memory (i.e. the ability to remember new information, at least as measured in the short-term) with increasing time since FMP while controlling for age. It would be useful to replicate this in further large studies and with a greater number of repeated measurements over a wider age span to determine whether this decline is temporary or permanent.

## Supplementary information

**Additional file 1: Supplementary Text.** – Analysis strategy. **Supplementary Table 1.** - Longitudinal analysis of cognitive function around menopause: results for reproductive age and chronological age (*N* = 2402–2408). **Supplementary Table 2.** - Longitudinal analysis of cognitive function by anti-Müllerian, follicle-stimulating and luteinizing hormone around menopause (*N* = 2209–2213). **Supplementary Table 3.** - Mean baseline standardised cognitive function for women who did and did not participate in all three assessment clinics. **Supplementary Table 4.** -. Mean standardised cognitive function from second clinic for women who did not participate in first assessment clinic. **Supplementary Table 5.** - Longitudinal analysis of cognitive function around menopause in women who participated in all three assessment clinics: results for reproductive age and chronological age (*N* = 1385–1386). **Supplementary Table 6.** - Longitudinal analysis of cognitive function by anti-Müllerian, follicle-stimulating and luteinizing hormone around menopause in women who participated in all three assessment clinics (*N* = 1348). **Supplementary Table 7.** - Percentage change in cognitive function by reproductive and chronological age.

## Data Availability

The datasets analysed during the current study and all ALSPAC data are available for analyses by any scientist upon application. Full details can be found at: http://www.bristol.ac.uk/alspac/researchers/access/. For analysis script, please contact the corresponding authors at fanny.kilpi@bristol.ac.uk.

## Abbreviations

ALSPAC: Avon longitudinal study of parents and children
AMH: Anti-Müllerian hormone
BIC: Bayesian information criteria
CI: Confidence interval
CSE: Certificate of secondary education
FMP: Final menstrual period
FSH: Follicle-stimulating hormone
HRT: Hormone replacement therapy
MAR: Missing-at-random
LH: Luteinizing hormone
SD: Standard deviation
STRAW: Stages of reproductive aging workshop
SWAN: Study of women’s health across the nation
