# Supplementary material for: Changes in six domains of cognitive function with reproductive and chronological ageing and sex hormones: a longitudinal study in 2411 UK mid-life women
Source: BMC Womens Health. 2020 Aug 14;20:177. doi: 10.1186/s12905-020-01040-3 (PMC7427852; doi:10.1186/s12905-020-01040-3)
Supplement: Supplementary file 1 — Additional file 1: Supplementary Text. – Analysis strategy. Supplementary Table 1. - Longitudinal analysis of cognitive function around menopause: results for reproductive age and chronological age (N = 2402–2408). Supplementary Table 2. - Longitudinal analysis of cognitive function by anti-Müllerian, follicle-stimulating and luteinizing hormone around menopause (N = 2209–2213). Supplementary Table 3. - Mean baseline standardised cognitive function for women who did and did not participate in all three assessment clinics. Supplementary Table 4. -. Mean standardised cognitive function from second clinic for women who did not participate in first assessment clinic. Supplementary Table 5. - Longitudinal analysis of cognitive function around menopause in women who participated in all three assessment clinics: results for reproductive age and chronological age (N = 1385–1386). Supplementary Table 6. - Longitudinal analysis of cognitive function by anti-Müllerian, follicle-stimulating and luteinizing hormone around menopause in women who participated in all three assessment clinics (N = 1348). Supplementary Table 7. - Percentage change in cognitive function by reproductive and chronological age. [file 12905_2020_1040_MOESM1_ESM.docx]

**Supplementary Text**

**ANALYSIS STRATEGY**

In the main analyses, performance on each cognitive test throughout the follow up was analysed using multilevel linear regression models (MLM). We used multilevel linear regression models to examine: (i) change in cognitive function domains by reproductive age (years since FMP) and chronological age and compare the contributions of each of these over time and (ii) the association of LH, FSH and AMH with change in cognitive function. Multilevel models allow all women with at least one cognitive function assessment to be included in analyses under a missing-at-random (MAR) assumption and take account of the correlation between repeat measurements. As we only had up to three measurements in each woman, we had to assume any change with reproductive or chronological age were linear. We modelled cognitive function domains in standard deviation (SD) units, using the mean from the first clinic assessment and the estimated between-individual SD derived from the fully adjusted model that included fieldworker effects, practice effects, reproductive and chronological age, education and age at first pregnancy.

The Bayesian Information Criteria (BIC) was used to assess and compare how well reproductive and chronological age explained variation in cognitive function. BIC penalises additional parameters in a model, and a smaller BIC reflects a better fit to the data. The models for the reproductive/chronological age analyses were as follows: (1) reproductive age as time scale, (2) reproductive age adjusted for practice effects, (3) chronological age as time scale (centred at 50), (4) chronological age adjusted for practice effects, (5) reproductive and chronological age mutually adjusted, (6) reproductive and chronological age mutually adjusted and adjusted for practice effects, and (7) reproductive and chronological age mutually adjusted and adjusted for practice effects, baseline education and age at first pregnancy (centred at 26). All models also controlled for fieldworker effects and whether the woman had had their FMP, the latter added as an interaction term with chronological age. Each of the models also included a random slope for reproductive and/or chronological age when they were included in the fixed part of the model.

We examined how time-varying LH, FSH and AMH were associated with cognitive function in a simple model adjusted for fieldworker effects, and in the fully adjusted model further including chronological age (with a random slope), practice effects, education and age at first pregnancy. FSH, LH and AMH were added as time-varying exposures, and their relationship to cognitive function was modelled as linear.

As sensitivity analyses, we compared baseline cognitive function scores by extent of follow-up participation to examine whether selective follow-up could bias the results. We repeated the main analyses and the hormone analyses in a sample restricted to women who participated in all three clinics.

Lastly, we studied differences in the extent of improvement by practice at pre-, peri- and post-menopause. We tested whether the interaction between practice effects (with a random slope) and menopausal stage improved model fit in a model with chronological age, education and age at first pregnancy using log likelihood tests.

Additionally, we compared our results with those of an independent UK study (Whitehall II) that examined ageing and cognition.(6) To make the results comparable, effect estimates of the present study were rescaled by dividing estimates on the original scale of the test by the full test range and multiplying by 100 to reflect a percentage difference on the range of each specific test.

**Supplementary Table 1. Longitudinal analysis of cognitive function around menopause: results for reproductive age and chronological age (N=2402-2408).**

|  | **Years since FMP (per 10 years) †** | **Chronological age**  **before FMP (per 10 years) ‡** | **Chronological age**  **after FMP (per 10 years) ‡** | **BIC** |
| --- | --- | --- | --- | --- |
|  | SD change (95% CI) | SD change (95% CI) | SD change (95% CI) |  |
| **Domain (cognitive test)** |  |  |  |  |
| **Verbal episodic memory**  **(immediate logical memory)** |  |  |  |  |
| (1) reproductive age* | 0.15 (-0.02, 0.32) |  |  | 19132 |
| (2) reproductive age* + practice effect | -0.03 (-0.20, 0.14) |  |  | 18831 |
| (3) chronological age* |  | 0.49 (0.30, 0.69) | 0.37 (0.23, 0.51) | 19121 |
| (4) chronological age* + practice effect |  | 0.07 (-0.13, 0.27) | 0.09 (-0.05, 0.23) | 18860 |
| (5) reproductive* + chronological age* | -0.31 (-0.53, -0.10) | 0.54 (0.34, 0.74) | 0.62 (0.40, 0.84) | 19106 |
| (6) reproductive* + chronological age* + practice effect | -0.25 (-0.47, -0.04) | 0.12 (-0.09, 0.32) | 0.29 (0.07, 0.51) | 18851 |
| (7) reproductive* + chronological age* + practice effect* + education + age at 1st pregnancy | -0.15 (-0.35, 0.06) | -0.21 (-0.42, 0.00) | -0.00 (-0.23, 0.22) | 18652 |
| **Verbal episodic memory**  **(delayed logical memory)** |  |  |  |  |
| (1) reproductive age* | 0.18 (0.01, 0.35) |  |  | 19137 |
| (2) reproductive age* + practice effect | -0.04 (-0.21, 0.13) |  |  | 18655 |
| (3) chronological age* |  | 0.55 (0.36, 0.74) | 0.43 (0.29, 0.57) | 19101 |
| (4) chronological age* + practice effect |  | 0.04 (-0.15, 0.23) | 0.08 (-0.06, 0.22) | 18675 |
| (5) reproductive* + chronological age* | -0.33 (-0.54, -0.12) | 0.60 (0.40, 0.80) | 0.68 (0.46, 0.90) | 19101 |
| (6) reproductive* + chronological age* + practice effect | -0.26 (-0.47, -0.06) | 0.08 (-0.11, 0.27) | 0.28 (0.07, 0.49) | 18680 |
| (7) reproductive* + chronological age* + practice effect + education + age at 1st pregnancy | -0.17 (-0.37, 0.03) | -0.23 (-0.44, -0.03) | -0.00 (-0.22, 0.21) | 18490 |
| **Working memory**  **(backward digit span)** |  |  |  |  |
| (1) reproductive age* | 0.17 (0.03, 0.30) |  |  | 16476 |
| (2) reproductive age* + practice effect | 0.06 (-0.08, 0.19) |  |  | 16371 |
| (3) chronological age* |  | 0.41 (0.26, 0.57) | 0.38 (0.26, 0.49) | 16445 |
| (4) chronological age* + practice effect |  | 0.20 (0.05, 0.36) | 0.22 (0.10, 0.34) | 16374 |
| (5) reproductive* + chronological age* | -0.08 (-0.24, 0.07) | 0.43 (0.27, 0.58) | 0.44 (0.28, 0.61) | 16459 |
| (6) reproductive* + chronological age* + practice effect | -0.06 (-0.22, 0.09) | 0.21 (0.06, 0.37) | 0.28 (0.11, 0.45) | 16388 |
| (7) reproductive* + chronological age* + practice effect + education + age at 1st pregnancy | 0.01 (-0.14, 0.16) | -0.04 (-0.21, 0.12) | 0.03 (-0.14, 0.21) | 16241 |
| **Processing speed**  **(digit symbol coding)** |  |  |  |  |
| (1) reproductive age* | -0.15 (-0.28, -0.02) |  |  | 13841 |
| (2) reproductive age* + practice effect | -0.42 (-0.55, -0.29) |  |  | 13478 |
| (3) chronological age* |  | 0.24 (0.12, 0.37) | -0.07 (-0.17, 0.03) | 13849 |
| (4) chronological age* + practice effect |  | -0.23 (-0.36, -0.10) | -0.45 (-0.56, -0.35) | 13487 |
| (5) reproductive* + chronological age* | -0.21 (-0.37, -0.06) | 0.19 (0.09, 0.30) | 0.07 (-0.07, 0.21) | 13860 |
| (6) reproductive* + chronological age* + practice effect | -0.25 (-0.40, -0.10) | -0.16 (-0.23, -0.09) | -0.26 (-0.40, -0.12) | 13492 |
| (7) reproductive* + chronological age* + practice effect + education + age at 1st pregnancy | -0.21 (-0.36, -0.06) | -0.37 (-0.51, -0.23) | -0.46 (-0.61, -0.31) | 13435 |
| **Verbal intelligence**  **(spot-the-word)** |  |  |  |  |
| (1) reproductive age* | 0.44 (0.31, 0.57) |  |  | 13991 |
| (2) reproductive age* + practice effect | 0.44 (0.30, 0.57) |  |  | 13999 |
| (3) chronological age* |  | 0.58 (0.45, 0.71) | 0.64 (0.53, 0.74) | 13897 |
| (4) chronological age* + practice effect |  | 0.74 (0.59, 0.88) | 0.77 (0.65, 0.88) | 13880 |
| (5) reproductive* + chronological age* | -0.05 (-0.22, 0.12) | 0.59 (0.46, 0.72) | 0.69 (0.53, 0.84) | 13912 |
| (6) reproductive* + chronological age* + practice effect | -0.06 (-0.23, 0.11) | 0.76 (0.61, 0.91) | 0.83 (0.66, 0.99) | 13894 |
| (7) reproductive* + chronological age* + practice effect + education + age at 1st pregnancy | 0.06 (-0.08, 0.21) | 0.28 (0.15, 0.41) | 0.33 (0.18, 0.48) | 13230 |
| **Verbal fluency**  **(same letter word)** |  |  |  |  |
| (1) reproductive age* | 0.23 (0.09, 0.36) |  |  | 14891 |
| (2) reproductive age* + practice effect | 0.05 (-0.09, 0.18) |  |  | 14720 |
| (3) chronological age* |  | 0.62 (0.48, 0.75) | 0.48 (0.37, 0.59) | 14797 |
| (4) chronological age* + practice effect |  | 0.31 (0.21, 0.42) | 0.26 (0.15, 0.37) | 14700 |
| (5) reproductive* + chronological age* | -0.14 (-0.30, 0.03) | 0.64 (0.50, 0.77) | 0.58 (0.42, 0.74) | 14813 |
| (6) reproductive* + chronological age* + practice effect | -0.12 (-0.28, 0.04) | 0.32 (0.21, 0.43) | 0.35 (0.19, 0.51) | 14716 |
| (8) reproductive* + chronological age* + practice effect + education + age at 1st pregnancy | -0.06 (-0.22, 0.09) | 0.16 (0.04, 0.28) | 0.14 (-0.02, 0.30) | 14483 |

Note: All models adjust for fieldworker effects and time-varying binary variable on whether final menstrual period had occurred.

* Asterix denotes variables which include a random slope in the model.

† Results refer to standard deviation difference in cognitive function score per 10 years change in reproductive age (years since final menstrual period).

‡ Results refer to standard deviation difference in cognitive function score per 10 years change in age (centred at 50).

BIC: Bayesian Information Criteria; FMP: final menstrual period

**Supplementary Table 2. Longitudinal analysis of cognitive function by anti-Müllerian, follicle-stimulating and luteinizing hormone around menopause (N=2209-2213).**

|  | **Hormone** | | |
| --- | --- | --- | --- |
| **Domain (cognitive test)** | **AMH** | **FSH** | **LH** |
| **Verbal episodic memory (immediate logical memory)** | SD difference (95% CI) | SD difference (95% CI) | SD difference (95% CI) |
| Unadjusted* | -0.05 (-0.11, 0.01) | 0.03 (-0.02, 0.08) | -0.01 (-0.06, 0.03) |
| Adjusted** | 0.03 (-0.04, 0.10) | -0.08 (-0.13, -0.02) | -0.08 (-0.13, -0.03) |
| **Verbal episodic memory (delayed logical memory)** |  |  |  |
| Unadjusted* | -0.08 (-0.15, -0.02) | 0.08 (0.03, 0.13) | 0.03 (-0.02, 0.08) |
| Adjusted** | 0.01 (-0.05, 0.08) | -0.04 (-0.09, 0.01) | -0.05 (-0.10, 0.00) |
| **Working memory (backward digit span)** |  |  |  |
| Unadjusted* | -0.07 (-0.13, -0.02) | 0.09 (0.05, 0.13) | 0.07 (0.03, 0.11) |
| Adjusted** | 0.00 (-0.05, 0.06) | 0.02 (-0.02, 0.06) | 0.02 (-0.02, 0.06) |
| **Processing speed (digit symbol coding)** |  |  |  |
| Unadjusted* | -0.02 (-0.06, 0.03) | 0.05 (0.02, 0.08) | 0.02 (-0.01, 0.05) |
| Adjusted** | 0.00 (-0.04, 0.04) | 0.04 (0.00, 0.07) | 0.02 (-0.01, 0.05) |
| **Verbal intelligence (spot-the-word)** |  |  |  |
| Unadjusted* | -0.08 (-0.12, -0.04) | 0.10 (0.07, 0.13) | 0.05 (0.02, 0.08) |
| Adjusted** | 0.02 (-0.03, 0.06) | 0.00 (-0.03, 0.04) | -0.01 (-0.04, 0.02) |
| **Verbal fluency (same letter word)** |  |  |  |
| Unadjusted* | -0.09 (-0.14, -0.04) | 0.11 (0.07, 0.14) | 0.07 (0.04, 0.11) |
| Adjusted** | 0.01 (-0.04, 0.05) | 0.01 (-0.03, 0.05) | 0.01 (-0.03, 0.04) |

Note: AMH: anti-Müllerian hormone, FSH: follicle-stimulating hormone, LH: luteinizing hormone, SD: standard deviation.

Results reflect SD difference in cognitive test score for one SD difference in hormone levels.

* Model adjusted for fieldworker effects

** Model adjusted for fieldworker effects, practice effects, age, education and age at first pregnancy.

**Supplementary Table 3. Mean baseline standardised cognitive function for women who did and did not participate in all three assessment clinics.**

| **Domain (cognitive test)** | N | Mean baseline standardised score | Mean difference (95% CI) |
| --- | --- | --- | --- |
| **Verbal episodic memory (immediate logical memory)** |  |  |  |
| All three assessment clinics | 1361 | -0.010 | ref |
| 1st and 2nd clinic | 241 | 0.057 | 0.07 (-0.15, 0.28) |
| 1st and 3rd clinic | 69 | 0.091 | 0.10 (-0.28, 0.48) |
| Only 1st assessment clinic | 278 | -0.022 | -0.01 (-0.22, 0.19) |
| **Verbal episodic memory (delayed logical memory)** |  |  |  |
| All three assessment clinics | 1353 | -0.008 | ref |
| 1st and 2nd clinic | 238 | 0.003 | 0.01 (-0.21, 0.23) |
| 1st and 3rd clinic | 69 | 0.051 | 0.06 (-0.32, 0.44) |
| Only 1st assessment clinic | 277 | 0.023 | 0.03 (-0.17, 0.23) |
| **Working memory (backward digit span)** |  |  |  |
| All three assessment clinics | 1360 | 0.030 | ref |
| 1st and 2nd clinic | 241 | 0.053 | 0.02 (-0.16, 0.20) |
| 1st and 3rd clinic | 69 | -0.412 | -0.44 (-0.76, -0.13) |
| Only 1st assessment clinic | 278 | -0.089 | -0.12 (-0.29,0.05) |
| **Processing speed (digit symbol coding)** |  |  |  |
| All three assessment clinics | 1353 | 0.022 | ref |
| 1st and 2nd clinic | 239 | -0.068 | -0.09 (-0.25, 0.07) |
| 1st and 3rd clinic | 69 | -0.215 | -0.24 (-0.51, 0.04) |
| Only 1st assessment clinic | 273 | 0.005 | -0.02 (-0.17, 0.13) |
| **Verbal intelligence (spot-the-word)** |  |  |  |
| All three assessment clinics | 1358 | 0.053 | ref |
| 1st and 2nd clinic | 241 | -0.097 | -0.15 (-0.33, 0.03) |
| 1st and 3rd clinic | 69 | -0.343 | -0.39 (-0.72, -0.07) |
| Only 1st assessment clinic | 276 | -0.093 | -0.15 (-0.32, 0.03) |
| **Verbal fluency (same letter word)** |  |  |  |
| All three assessment clinics | 1353 | 0.039 | ref |
| 1st and 2nd clinic | 239 | 0.002 | -0.04 (-0.20, 0.13) |
| 1st and 3rd clinic | 69 | -0.352 | -0.39 (-0.68, -0.10) |
| Only 1st assessment clinic | 275 | -0.103 | -0.14 (-0.30, 0.01) |

**Supplementary Table 4. Mean standardised cognitive function from second assessment clinic for women who did not participate in first clinic.**

| **Domain (cognitive test)** | N | Mean baseline standardised score | Mean difference |
| --- | --- | --- | --- |
| **Verbal episodic memory (immediate logical memory)** |  |  |  |
| 2nd and 3rd clinic | 167 | -0.414 | ref |
| Only 2nd assessment clinic | 104 | -0.312 | 0.10 (-0.30, 0.51) |
| **Verbal episodic memory (delayed logical memory)** |  |  |  |
| 2nd and 3rd clinic | 167 | -0.439 | ref |
| Only 2nd assessment clinic | 104 | -0.295 | 0.14 (-0.26, 0.54) |
| **Working memory**  **(backward digit span)** |  |  |  |
| 2nd and 3rd clinic | 167 | -0.214 | ref |
| Only 2nd assessment clinic | 104 | -0.163 | 0.05 (-0.24, 0.35) |
| **Processing speed**  **(digit symbol coding)** |  |  |  |
| 2nd and 3rd clinic | 166 | -0.239 | ref |
| Only 2nd assessment clinic | 104 | -0.202 | 0.04 (-0.25, 0.32) |
| **Verbal intelligence**  **(spot-the-word)** |  |  |  |
| 2nd and 3rd clinic | 166 | -0.285 | ref |
| Only 2nd assessment clinic | 104 | -0.283 | 0.00 (-0.31, 0.31) |
| **Verbal fluency**  **(same letter word)** |  |  |  |
| 2nd and 3rd clinic | 166 | -0.110 | ref |
| Only 2nd assessment clinic | 104 | 0.030 | 0.14 (-0.18, 0.46) |

**Supplementary Table 5.** **Longitudinal analysis of cognitive function around menopause in women who participated in all three assessment clinics: results for reproductive age and chronological age (N=1385-1386).**

|  | **Years since FMP (per 10 years) †** | **Chronological age**  **before FMP (per 10 years) ‡** | **Chronological age**  **after FMP (per 10 years) ‡** | BIC |
| --- | --- | --- | --- | --- |
|  | SD change (95% CI) | SD change (95% CI) | SD change (95% CI) |  |
| **Domain (cognitive test)** |  |  |  |  |
| **Verbal episodic memory**  **(immediate logical memory)** |  |  |  |  |
| (1) reproductive age* | 0.25 (0.03, 0.46) |  |  | 13716 |
| (2) reproductive age* + practice effect | -0.08 (-0.29, 0.14) |  |  | 13479 |
| (3) chronological age* |  | 0.64 (0.41, 0.88) | 0.48 (0.31, 0.66) | 13702 |
| (4) chronological age* + practice effect |  | 0.11 (-0.13, 0.35) | 0.10 (-0.08, 0.28) | 13508 |
| (5) reproductive* + chronological age* | -0.30 (-0.58, -0.02) | 0.69 (0.45, 0.94) | 0.71 (0.43, 0.98) | 13689 |
| (6) reproductive* + chronological age* + practice effect | -0.29 (-0.56, -0.02) | 0.17 (-0.07, 0.42) | 0.32 (0.05, 0.59) | 13498 |
| (7) reproductive* + chronological age* + practice effect* + education + age at 1st pregnancy | -0.18 (-0.44, 0.08) | -0.11 (-0.36, 0.15) | 0.01 (-0.26, 0.29) | 13387 |
| **Verbal episodic memory**  **(delayed logical memory)** |  |  |  |  |
| (1) reproductive age* | 0.28 (0.06, 0.50) |  |  | 13709 |
| (2) reproductive age* + practice effect | -0.12 (-0.33, 0.09) |  |  | 13328 |
| (3) chronological age* |  | 0.76 (0.53, 0.99) | 0.59 (0.42, 0.76) | 13661 |
| (4) chronological age* + practice effect |  | 0.11 (-0.12, 0.33) | 0.12 (-0.05; 0.30) | 13345 |
| (5) reproductive* + chronological age* | -0.41 (-0.69, -0.13) | 0.82 (0.58, 1.05) | 0.89 (0.62, 1.16) | 13658 |
| (6) reproductive* + chronological age* + practice effect | -0.40 (-0.67, -0.13) | 0.17 (-0.06, 0.41) | 0.42 (0.16, 0.68) | 13345 |
| (7) reproductive* + chronological age* + practice effect + education + age at 1st pregnancy | -0.30 (-0.56, -0.04) | -0.11 (-0.36, 0.13) | 0.11 (-0.16, 0.38) | 13239 |
| **Working memory**  **(backward digit span)** |  |  |  |  |
| (1) reproductive age* | 0.24 (0.06, 0.43) |  |  | 11767 |
| (2) reproductive age* + practice effect | 0.06 (-0.12, 0.24) |  |  | 11696 |
| (3) chronological age* |  | 0.50 (0.31, 0.68) | 0.48 (0.33, 0.63) | 11735 |
| (4) chronological age* + practice effect |  | 0.25 (0.06, 0.44) | 0.28 (0.13, 0.44) | 11696 |
| (5) reproductive* + chronological age* | -0.08 (-0.30, 0.13) | 0.50 (0.32, 0.69) | 0.54 (0.33, 0.76) | 11755 |
| (6) reproductive* + chronological age* + practice effect | -0.08 (-0.29, 0.14) | 0.25 (0.06, 0.45) | 0.34 (0.12, 0.56) | 11715 |
| (7) reproductive* + chronological age* + practice effect + education + age at 1st pregnancy | 0.00 (-0.21, 0.22) | 0.04 (-0.16, 0.24) | 0.10 (-0.13, 0.33) | 11652 |
| **Processing speed**  **(digit symbol coding)** |  |  |  |  |
| (1) reproductive age* | 0.01 (-0.16, 0.17) |  |  | 9665 |
| (2) reproductive age* + practice effect | -0.39 (-0.55, -0.23) |  |  | 9381 |
| (3) chronological age* |  | 0.45 (0.30, 0.60) | 0.09 (-0.03, 0.21) | 9647 |
| (4) chronological age* + practice effect |  | -0.14 (-0.30, 0.02) | -0.41 (-0.55, -0.28) | 9396 |
| (5) reproductive* + chronological age* | -0.17 (-0.34, 0.01) | 0.52 (0.39, 0.65) | 0.19 (0.14, 0.25) | 9650 |
| (6) reproductive* + chronological age* + practice effect | -0.68 (did not converge) | -0.01 (did not converge) | 0.03 (did not converge) | did not converge |
| (7) reproductive* + chronological age* + practice effect + education + age at 1st pregnancy | -0.22 (-0.41, -0.04) | -0.19 (-0.36, -0.03) | -0.36 (-0.55, -0.17) | 9383 |
| **Verbal intelligence**  **(spot-the-word)** |  |  |  |  |
| (1) reproductive age* | 0.43 (0.26, 0.59) |  |  | 9639 |
| (2) reproductive age* + practice effect | 0.46 (0.28, 0.63) |  |  | 9646 |
| (3) chronological age* |  | 0.43 (0.27, 0.58) | 0.57 (0.44, 0.69) | 9604 |
| (4) chronological age* + practice effect |  | 0.69 (0.51, 0.87) | 0.79 (0.64, 0.94) | 9581 |
| (5) reproductive* + chronological age* | -0.03 (-0.25, 0.18) | 0.41 (0.26, 0.57) | 0.60 (0.41, 0.78) | 9619 |
| (6) reproductive* + chronological age* + practice effect | -0.04 (-0.26, 0.17) | 0.69 (0.51, 0.87) | 0.83 (0.62, 1.03) | 9597 |
| (7) reproductive* + chronological age* + practice effect + education + age at 1st pregnancy | 0.06 (-0.14, 0.26) | 0.28 (0.11, 0.45) | 0.34 (0.15, 0.53) | 9213 |
| **Verbal fluency**  **(same letter word)** |  |  |  |  |
| (1) reproductive age* | 0.26 (0.09, 0.43) |  |  | 10512 |
| (2) reproductive age* + practice effect | -0.02 (-0.19, 0.15) |  |  | 10382 |
| (3) chronological age* |  | 0.62 (0.46, 0.78) | 0.58 (0.44, 0.71) | 10436 |
| (4) chronological age* + practice effect |  | did not converge | did not converge | - |
| (5) reproductive* + chronological age* | did not converge | did not converge | did not converge | - |
| (6) reproductive* + chronological age* + practice effect | -0.22 (-0.42, -0.01) | 0.28 (0.22, 0.34) | 0.43 (0.24, 0.63) | 10384 |
| (8) reproductive* + chronological age + practice effect + education + age at 1st pregnancy | -0.18 (-0.38, 0.03) | 0.15 (-0.03, 0.33) | 0.23 (0.02, 0.44) | 10242 |

Note: All models adjust for fieldworker effects and time-varying binary variable on whether final menstrual period had occurred.

* Asterix denotes variables which include a random slope in the model.

† Results refer to standard deviation difference in cognitive function score per 10 years change in reproductive age (years since final menstrual period).

‡ Results refer to standard deviation difference in cognitive function score per 10 years change in age.

BIC: Bayesian Information Criteria; FMP: final menstrual period

**Supplementary Table 6.** **Longitudinal analysis of cognitive function by anti-Müllerian, follicle-stimulating and luteinizing hormone around menopause in women who participated in all three assessment** **clinics (N=1348).**

|  | **Hormone** | | |
| --- | --- | --- | --- |
| **Domain (cognitive test)** | **AMH** | **FSH** | **LH** |
| **Verbal episodic memory**  **(immediate logic memory)** | SD difference (95% CI) | SD difference (95% CI) | SD difference (95% CI) |
| Unadjusted* | -0.04 (-0.12. 0.03) | 0.05 (-0.00, 0.11) | -0.01 (-0.07, 0.04) |
| Adjusted** | 0.04 (-0.03. 0.12) | -0.06 (-0.12, 0.01) | -0.08 (-0.14, -0.02) |
| **Verbal episodic memory**  **(delayed logic memory)** |  |  |  |
| Unadjusted* | -0.10 (-0.17, -0.02) | 0.11 (0.05, 0.16) | 0.04 (-0.01, 0.10) |
| Adjusted** | 0.02 (-0.06, 0.09) | -0.03 (-0.09, 0.04) | -0.04 (-0.10, 0.01) |
| **Working memory**  **(digits backwards)** |  |  |  |
| Unadjusted* | -0.08 (-0.14, -0.02) | 0.10 (0.05, 0.14) | 0.06 (0.01, 0.10) |
| Adjusted** | 0.00 (-0.06, 0.06) | 0.02 (-0.03, 0.07) | 0.01 (-0.04, 0.06) |
| **Processing speed**  **(digit symbol)** |  |  |  |
| Unadjusted* | -0.01 (-0.06, 0.04) | 0.06 (0.03, 0.10) | 0.03 (-0.01, 0.06) |
| Adjusted** | 0.03 (-0.02, 0.08) | 0.02 (-0.02, 0.06) | 0.01 (-0.03, 0.04) |
| **Verbal intelligence**  **(spot-the-word)** |  |  |  |
| Unadjusted* | -0.06 (-0.10, -0.01) | 0.08 (0.04, 0.11) | 0.03 (-0.00, 0.06) |
| Adjusted** | 0.01 (-0.04, 0.06) | 0.01 (-0.03, 0.04) | -0.02 (-0.05, 0.02) |
| **Verbal fluency**  **(same letter word)** |  |  |  |
| Unadjusted* | -0.08 (-0.14, -0.03) | 0.12 (0.08, 0.16) | 0.07 (0.03, 0.11) |
| Adjusted** | 0.02 (-0.04, 0.07) | 0.02 (-0.02, 0.07) | 0.01 (-0.03, 0.05) |

Note: AMH: anti-Müllerian hormone, FSH: follicle-stimulating hormone, LH: luteinizing hormone. Results reflect standard deviation difference in cognitive test score for one standard deviation difference in hormone levels.

* Model adjusted for fieldworker effects

** Model adjusted for fieldworker effects, practice effects, age, education and age at first pregnancy.

**Supplementary Table 7. Percentage change in cognitive function by reproductive and chronological age.**

|  | **Years since FMP (per 10 years)** | **Chronological age before FMP (per 10 years)** | **Chronological age after FMP (per 10 years)** |
| --- | --- | --- | --- |
| **Domain**  **(cognitive test)** | Percentage change (95% CI) | Percentage change (95% CI) | Percentage change (95% CI) |
| **Verbal episodic memory (immediate logical memory)** | -1.28 (-3.05, 0.50) | -1.83 (-3.66, 0.01) | -0.02 (-1.95, 1.92) |
| **Verbal episodic memory (delayed logical memory)** | -1.58 (-3.43, 0.29) | -2.17 (-4.05, -0.29) | -0.04 (-2.05, 1.97) |
| **Working memory**  **(backward digit span)** | 0.13 (-1.83, 2.10) | -0.52 (-2.64, 1.61) | 0.44 (-1.80, 2.68) |
| **Processing speed**  **(digit symbol coding)** | -1.89 (-3.25, -0.52) | -3.35 (-4.62, -2.08) | -4.15 (-5.54, -2.76) |
| **Verbal intelligence**  **(spot-the-word)** | 0.60 (-0.80, 2.00) | 2.64 (1.37, 3.90) | 3.12 (1.73, 4.51) |
| **Verbal fluency**  **(same letter word)** | -0.64 (-2.23, 0.96) | 1.63 (0.40, 2.86) | 1.42 (-0.20, 3.05) |

Note: All models adjust for reproductive age, chronological age, fieldworker effects, education, age at first pregnancy, and time-varying binary variable on whether final menstrual period had occurred.
